# Postprandial metabolism of apolipoproteins B48, B100, C-III, and E in humans with *APOC3* loss-of-function mutations

**DOI:** 10.1172/jci.insight.160607

**Published:** 2022-10-10

**Authors:** Marja-Riitta Taskinen, Elias Björnson, Niina Matikainen, Sanni Söderlund, Joel Rämö, Mari-Mia Ainola, Antti Hakkarainen, Carina Sihlbom, Annika Thorsell, Linda Andersson, Per-Olof Bergh, Marcus Henricsson, Stefano Romeo, Martin Adiels, Samuli Ripatti, Markku Laakso, Chris J. Packard, Jan Borén

**Affiliations:** 1 Clinical and Molecular Medicine, Research Programs Unit, Faculty of Medicine, University of Helsinki, Helsinki, Finland.; 2 Department of Molecular and Clinical Medicine, Institute of Medicine, University of Gothenburg, Sweden.; 3 Endocrinology, Abdominal Center, Helsinki University Hospital, Helsinki, Finland.; 4 Institute for Molecular Medicine Finland, Helsinki Institute of Life Science (HiLIFE), University of Helsinki, Helsinki, Finland.; 5 Broad Institute of MIT and Harvard, Cambridge, Massachusetts, USA.; 6 HUS Medical Imaging Center, Radiology, Helsinki University Hospital, University of Helsinki, Finland.; 7 Proteomics Core Facility, University of Gothenburg, Gothenburg, Sweden.; 8 Department of Public Health, Clinicum, Faculty of Medicine, University of Helsinki, Helsinki, Finland.; 9 Institute of Clinical Medicine, Internal Medicine, University of Eastern Finland, Kuopio, Finland.; 10 Department of Medicine, Kuopio University Hospital, Kuopio, Finland.; 11 Institute of Cardiovascular and Medical Sciences, University of Glasgow, Glasgow, United Kingdom.; 12 Sahlgrenska University Hospital, Gothenburg, Sweden.

**Keywords:** Metabolism, Lipoproteins

## Abstract

**Background:**

Apolipoprotein C-III (apoC-III) is a regulator of triglyceride (TG) metabolism, and due to its association with risk of cardiovascular disease, is an emergent target for pharmacological intervention. The impact of substantially lowering apoC-III on lipoprotein metabolism is not clear.

**Methods:**

We investigated the kinetics of apolipoproteins B48 and B100 (apoB48 and apoB100) in chylomicrons, VLDL_1_, VLDL_2_, IDL, and LDL in patients heterozygous for a loss-of-function (LOF) mutation in the *APOC3* gene. Studies were conducted in the postprandial state to provide a more comprehensive view of the influence of this protein on TG transport.

**Results:**

Compared with non-LOF variant participants, a genetically determined decrease in apoC-III resulted in marked acceleration of lipolysis of TG-rich lipoproteins (TRLs), increased removal of VLDL remnants from the bloodstream, and substantial decrease in circulating levels of VLDL_1_, VLDL_2_, and IDL particles. Production rates for apoB48-containing chylomicrons and apoB100-containing VLDL_1_ and VLDL_2_ were not different between LOF carriers and noncarriers. Likewise, the rate of production of LDL was not affected by the lower apoC-III level, nor were the concentration and clearance rate of LDL-apoB100.

**Conclusion:**

These findings indicate that apoC-III lowering will have a marked effect on TRL and remnant metabolism, with possibly significant consequences for cardiovascular disease prevention.

**Trial registration:**

ClinicalTrials.gov NCT04209816 and NCT01445730.

**Funding:**

Swedish Heart-Lung Foundation, Swedish Research Council, ALF grant from the Sahlgrenska University Hospital, Novo Nordisk Foundation, Sigrid Juselius Foundation, Helsinki University Hospital Government Research funds, Finnish Heart Foundation, and Finnish Diabetes Research Foundation.

## Graphical abstract

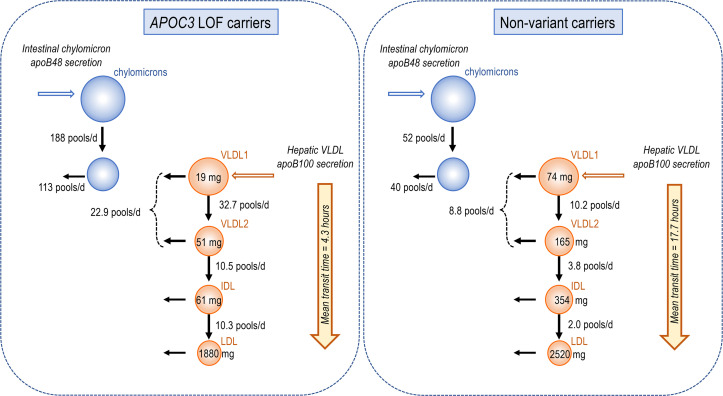

## Introduction

Apolipoprotein C-III (apoC-III) is a small protein (comprising 70 amino acid residues) found on triglyceride-rich lipoproteins (TRLs) — chylomicrons and VLDL — that appears to be an important regulator of their intravascular metabolism (1–3). It is synthesized in the liver and intestine and in the circulation transfers freely between lipoprotein particles, mainly TRL and HDL, but it is also present on LDL (1, 4). Genetic studies provide strong evidence linking variation in the *APOC3* gene that encodes apoC-III with altered plasma triglyceride (TG) levels and risk of atherosclerosis (1–3, 5–9).

The existence of a strong, positive association between plasma apoC-III and TG levels has been appreciated for many years and raised the possibility that apoC-III was a key determinant of TRL metabolism. A mechanistic basis for this relationship emerged when it was discovered that apoC-III acted as an inhibitor of lipoprotein lipase (LPL), the main enzyme responsible for TG hydrolysis in both chylomicrons and VLDL (10–16). Indeed, the rate of TRL lipolysis was linked to the ratio of apoC-II (another small apolipoprotein that is an activator of LPL) to apoC-III on the particle surface (1–3). In the process of TRL lipolysis, cholesterol-enriched remnants are generated, and recent reports have identified these remnant particles as independent risk factors for atherosclerotic cardiovascular disease (ASCVD) (17–22). According to current concepts, a slower lipolysis rate, due for example to higher apoC-III, favors remnant formation. There is evidence also, mainly in animal models, that a further action of apoC-III is to slow the rate of remnant removal by the liver, thereby prolonging the residence time of these particles in the circulation (1, 4, 13, 18, 23). The plasma level of apoC-III is determined by its rates of synthesis and catabolism, and human metabolic studies have shown that it is the apoC-III production rate that is a key determinant of plasma TG (24–27). ApoC-III may also have an intrahepatic function in that increased secretion of apoC-III has been reported to stimulate VLDL production in cell models (28).

Rare loss-of-function (LOF) variants in *APOC3* have been identified and shown to confer low TG levels (1, 5–9). One, the rs76353203 variant, introduces a stop codon in the signal peptide (R19X), preventing synthesis of the apoC-III protein (29). Interestingly, 5% of the Lancaster Amish are heterozygous carriers for this null mutation (29). Another, the rs138326449 variant, is a splice variant (IVS2+1G-A) of *APOC3* intron 2 (30). As compared with noncarriers of *APOC3* mutations, heterozygotes for these 2 mutations have mean reductions of 44% in nonfasting TG levels (7). The impact of the R19X mutation has been examined in a kinetic study in heterozygotes for the null variant and their unaffected siblings (31). It was found that in the fasting state the genetically determined low apoC-III concentration led to accelerated delipidation rates for VLDL and more rapid conversion to LDL.

The present study examined broader effects of apoC-III on TG metabolism by investigating in *APOC3* LOF carriers in the postprandial state. Our objective was to elucidate the regulatory role of apoC-III in this more physiologically relevant situation and thereby gain better insight into the potential for therapeutic interventions targeted at apoC-III to alter circulating levels of TRLs and their remnants and so potentially protect against cardiovascular disease (5–9). Our approach allows the derivation of production and clearance rates of intestinally derived apolipoprotein B48–lipoproteins (apoB48-lipoproteins) and liver-derived apoB100-containing lipoproteins, integrating the dynamics of TRL metabolism in the fed state with continuous hepatic secretion of lipoproteins. The kinetics of apoC-III and apoE were also investigated in *APOC3* LOF heterozygous carriers.

## Results

Key characteristics of the participant groups who participated in the kinetic study are shown in Table 1, and a broader range of cardiometabolic variables is provided in Supplemental Tables 1 and 2; supplemental material available online with this article; https://doi.org/10.1172/jci.insight.160607DS1 The *APOC3* LOF variant carriers were, after matching for age and BMI, similar in body fat distribution, liver fat content, and glycemic status to the non-LOF variant carriers (Table 1). Standard liver function test results were normal in both groups (Supplemental Table 1). The lipid and lipoprotein profiles were as expected for heterozygous carriers of an *APOC3* LOF variant. Plasma levels of apoC-III were 66% lower in the *APOC3* LOF carriers compared with nonvariant carriers (Table 1) (*P* < 0.0001). Plasma TG was also significantly lower by 57%, as was the fasting apoB48 level (by 71%, *P =* 0.025). In contrast, mean LDL-C and total plasma apoB were virtually the same in the 2 groups. There were no differences in other lipid-related variables, such as HDL-C, ApoAI, ApoA5, ApoE, and ANGPTL3 (Table 1). No between-group differences were seen in biomarkers of TG and fatty acid metabolism, including free fatty acid levels, β-hydroxybutyrate (a marker of fatty acid oxidation), and the activities of LPL and hepatic lipase.

TGs and phospholipids, specifically phosphatidylcholines, were subjected to lipidomic analysis to ascertain if alterations in apoB metabolism altered their fatty acid composition. No systematic differences were detected between the *APOC3* LOF carriers and noncarriers in the fatty acid species present in chylomicrons, VLDL_1_, VLDL_2_, IDL, or LDL (Supplemental Figure 2, A and B).

### ApoB100, apoB48, and TG metabolism in APOC3 LOF heterozygous carriers.

**Figure 1:**
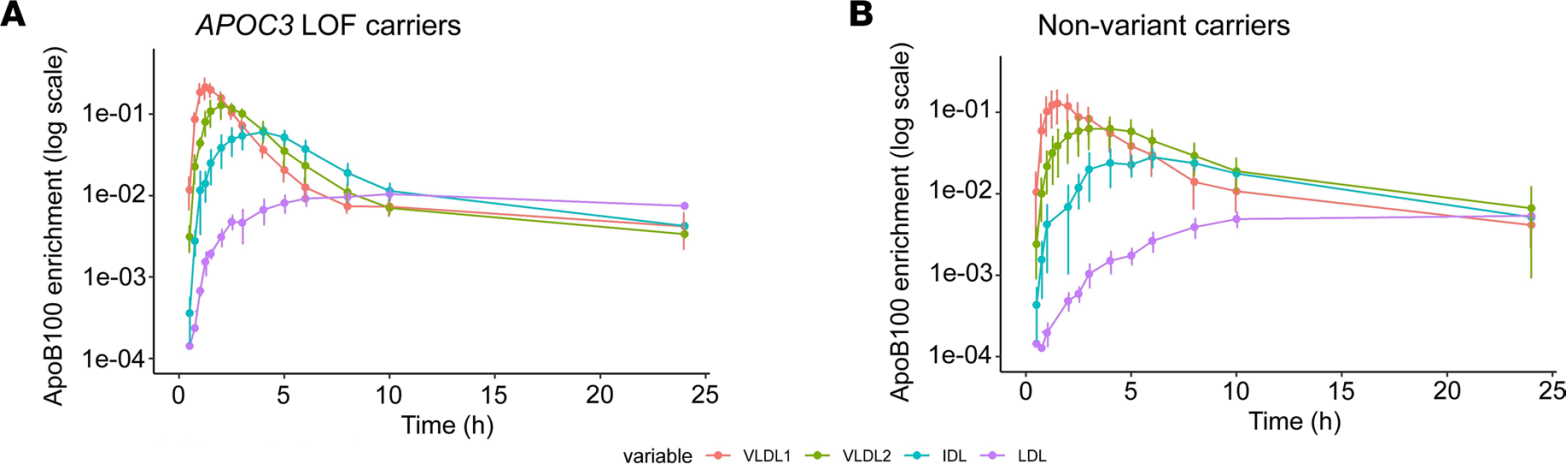
Enrichment curves for VLDL_1_, VLDL_2_, IDL, and LDL apoB100 in patients with *APOC3* LOF variants and nonvariant carriers. Tracer/tracee ratios (data points are means and vertical lines represent standard deviations) are presented for apoB100 in each lipoprotein density range. Time in hours is from tracer administration. Solid line refers to the mean of the model fits.

Tracers of deuterated leucine and glycerol were used to determine the kinetic properties of apoB100, apoB48, and TG in these patients. The total set of lipoprotein concentration and protein enrichment data used as input to the compartmental model is shown in Supplemental Figure 1. A good fit of the model to the observed data was obtained (Supplemental Figure 1), and production, transfer, and clearance rates for lipoprotein classes were derived as set out in Table 2. To illustrate the impact of the *APOC3* LOF variants on the flow of apoB100 down the VLDL to LDL delipidation cascade, mean enrichment curves for VLDL_1_, VLDL_2_, IDL, and LDL apoB100 are presented in Figure 1. Here it can be seen that, compared with nonvariant carriers, *APOC3* LOF carriers exhibited a very rapid transfer of apoB100 (and hence lipoprotein particles since each particle has 1 integral apoB protein) from VLDL_1_ to VLDL_2_, from VLDL_2_ to IDL, and finally from IDL to LDL. The derived fractional transfer rates (FTRs) between these fractions reflected this metabolic behavior (Table 2); FTRs were increased significantly in the *APOC3* LOF carriers by about 3-fold compared with the nonvariant carriers. Similarly, the overall fractional clearance rates (FCRs) for VLDL_1_, VLDL_2_, and IDL apoB100 were increased by approximately the same degree. Total clearance from a density interval (reflected in the FCR) is a combination of transfer to the next component in the delipidation chain (lipolysis) and direct clearance of the apoB-containing particle from the circulation (fractional direct clearance rate, FDCR). It was noteworthy that the FDCRs for VLDL_1_ apoB100 and for total VLDL apoB100 were significantly higher in the *APOC3* LOF carriers compared with nonvariant carriers (Table 2). Further key findings were that the FCR for LDL apoB100 did not differ between the 2 groups and that the production rate for VLDL_1_ apoB100, direct production rate for VLDL_2_ apoB100, and total input of apoB into IDL plus LDL was comparable in *APOC3* LOF carriers versus nonvariant carriers (Table 2 and Supplemental Table 3). There was a nominally significant difference noted in direct LDL apoB100 synthesis (Supplemental Table 3).

TG kinetics in VLDL fractions revealed similar between-group differences as seen for apoB100 (Table 2). The FTR for VLDL_1_ to VLDL_2_ conversion, the VLDL_1_-TG FDCR, and the overall FCRs for VLDL_1_ and VLDL_2_ were 2- to 3-fold higher in *APOC3* LOF carriers. Again, production rates for TG in VLDL_1_ and VLDL_2_ were similar in the 2 groups.

**Figure 2:**
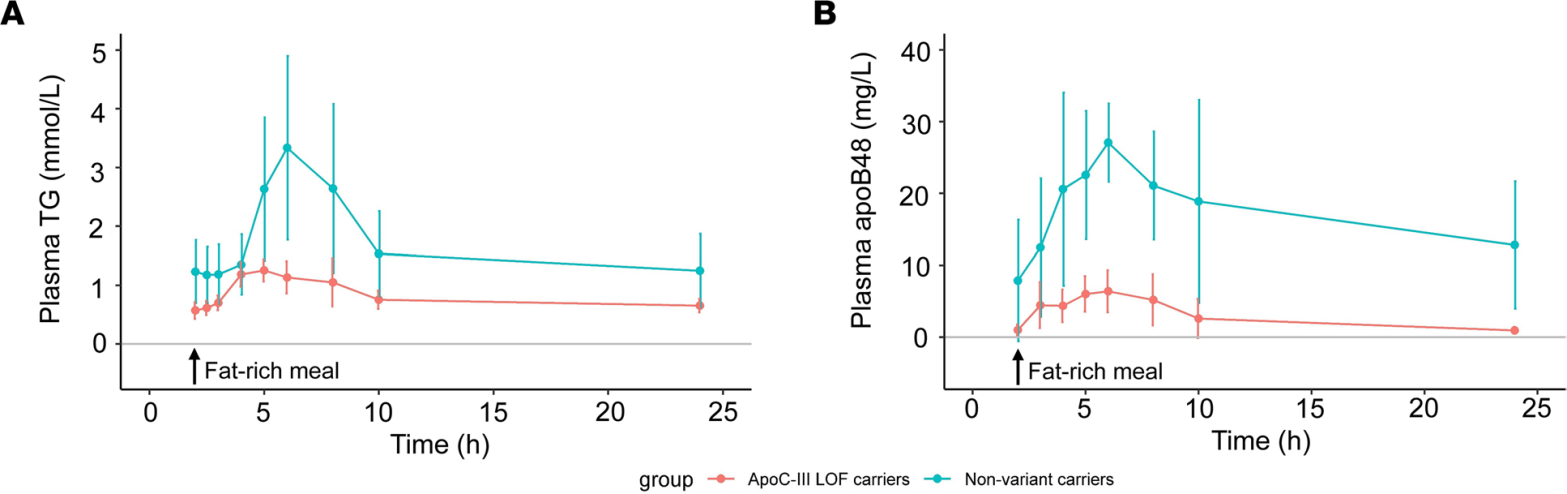
Postprandial lipemia responses in patients with *APOC3* LOF variants and nonvariant carriers. Data show the rise in plasma apoB48 and plasma TG after a standard fat-rich meal (data points are mean values and vertical bars present standard deviations). Time in hours is from tracer administration. The standard fat-rich meal was consumed at the 2-hour time point after tracer administration. Turquoise color refers to the nonvariant carrier group, and red color refers to the *APOC3* LOF carriers.

Metabolism of apoB48-containing particles in the chylomicron and VLDL density ranges was altered in the *APOC3* LOF carriers compared with nonvariant carriers (Figure 2, Table 2, and Supplemental Figure 1). There was a remarkable reduction in postprandial lipemia (AUC) in response to the standard fat-rich meal in the *APOC3* LOF carriers compared with nonvariant carriers (Table 1 and Figure 2) that was evident for both plasma TG (AUC reduced by 55%) and plasma apoB48 (AUC reduced by 76%). In line with the changes seen for VLDL apoB100-containing particles, overall clearance rates for chylomicrons and apoB48-containing VLDL_1_ and VLDL_2_ were 2- to 3-fold higher in the *APOC3* LOF carriers relative to nonvariant carriers. These differences were nominally significant (*P* < 0.05) with the exception of the VLDL_1_ apoB48 FCR and the TG-apoB48 VLDL_1_ FCR, which showed a trend of approximately the same magnitude. Production rates of apoB48-containing particles either in the basal (fasted) state or postprandially following the test meal were not different between the 2 groups.

**Figure 3:**
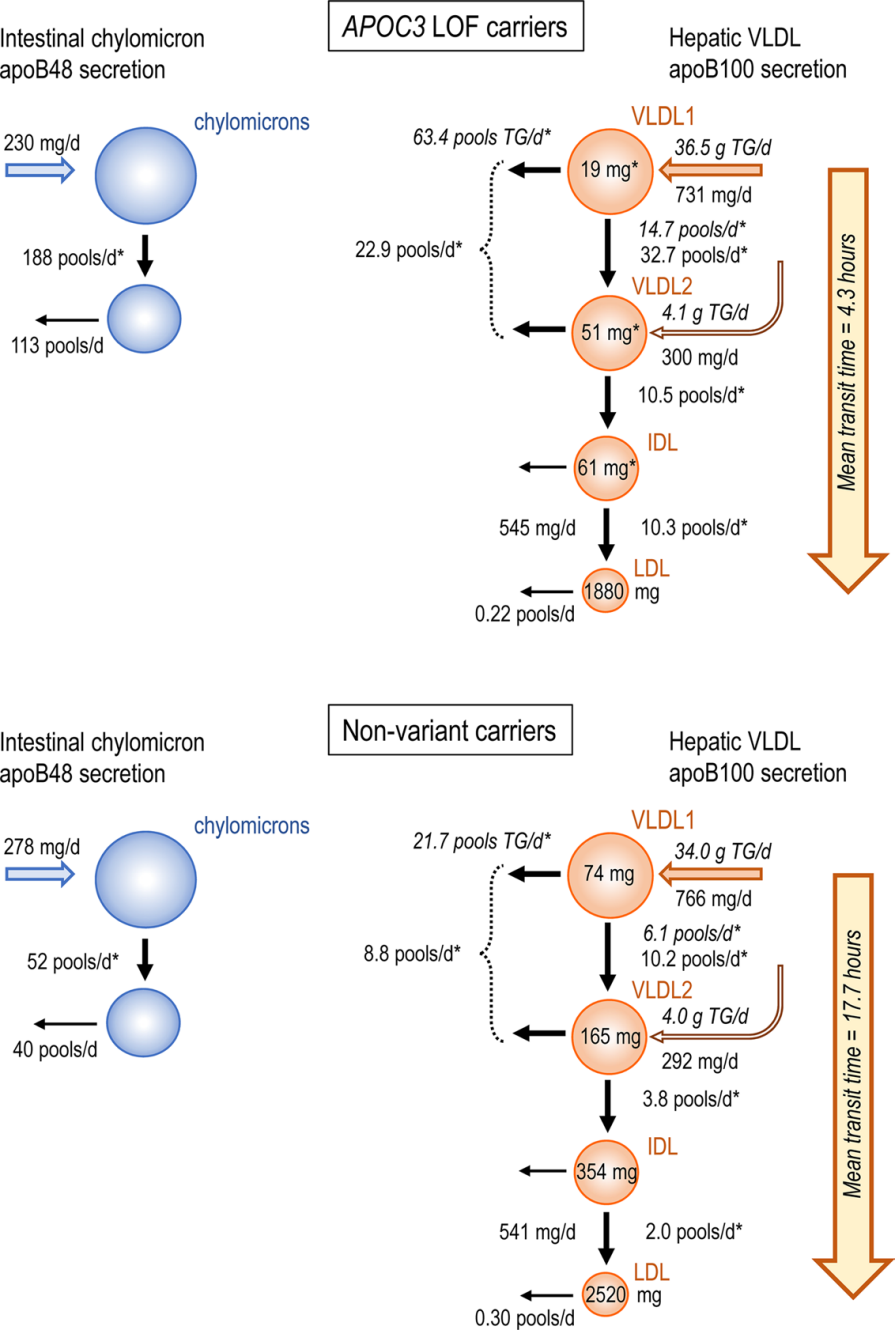
Flowchart showing key kinetic parameter differences in *APOC3* LOF carriers and nonvariant carriers. Data are mean values for each group of patients. Production rates are given in mg/d and FTRs and FDCRs in pools/d. The plasma pool of each apoB100-containing lipoprotein class (VLDL_1_, VLDL_2_, IDL, and LDL) is given (mg) within the appropriate circle. ApoB48 and apoB100 kinetic rate constants are in upright text; kinetic rate constants for TG are in italics. The mean transit time was calculated as the sum of the residence times for VLDL_1_, VLDL_2_, and IDL apoB-containing particles. Residence time is the reciprocal of the overall FCR; e.g., for VLDL_1_ apoB100 in nonvariant carriers the FCR is 13.1 pools/24 hours (Table 2), which gives a residence time of 24/13.1 = 1.83 hours. Asterisks indicate significant differences between *APOC3* LOF variants versus nonvariant carriers. *P* values are from group comparison using Mann-Whitney *U* test.

Figure 3 presents a schematic flowchart summarizing key attributes of apoB metabolism in *APOC3* LOF carriers compared with nonvariant carriers. Production rates for intestinally derived apoB48-containing chylomicrons (CM-apoB48) after a meal, and for apoB100-containing VLDL_1_ and VLDL_2_ released from the liver, appeared not to be affected in the *APOC3* LOF carriers. The substantially and significantly lower circulating pool sizes for VLDL_1_ apoB100 (74%), VLDL_2_ apoB100 (69%), and IDL apoB100 (83%) in the *APOC3* LOF carriers (Supplemental Table 3) were the result of enhanced clearance both down the delipidation chain — lipolysis rates were several-fold higher — and by direct catabolism of VLDL apoB100-containing particles. Higher lipolysis and overall clearance rates were also seen for CM-apoB48 (although the difference in direct chylomicron clearance did not reach significance). Estimation of the total transit time for an apoB100-containing VLDL_1_ particle to become LDL was 4.3 hours in *APOC3* LOF carriers versus 17.7 hours in nonvariant carriers (total transit time was taken as the sum of the residence times for VLDL_1_, VLDL_2_, and IDL apoB100-containing particles: residence time is the reciprocal of the FCR). Likewise, the mean residence time for a chylomicron particle (reciprocal of CM-apoB48 FCR in Table 2) was 7.7 minutes in *APOC3* LOF carriers compared with 28 minutes in nonvariant carriers.

### ApoC-III and apoE metabolism in APOC3 LOF heterozygotes.

Kinetic parameters were determined for apoC-III and apoE using enrichment data and individual compartmental models for each protein (Table 3). In the *APOC3* LOF carriers, the circulating pool size of apoC-III was reduced in line with the lower plasma concentration for this apolipoprotein. This difference compared with nonvariant carriers was attributable to both a 57% lower production rate and a 72% higher tractional clearance rate for the protein. In contrast, there were no differences in the rates of production and clearance of apoE (Table 3).

## Discussion

This detailed metabolic investigation in patients with genetically determined lower apoC-III was designed to uncover the regulatory actions that this apolipoprotein exerts on TG transport in humans. It was found that patients heterozygous for *APOC3* LOF variants exhibited marked and highly specific alterations in chylomicron and VLDL kinetics. First, the degree of postprandial lipemia following a standardized fat-rich meal was greatly reduced in *APOC3* LOF carriers. Second, on average, the overall clearance rates of VLDL_1_, VLDL_2_, IDL, and chylomicrons were about 3-fold higher in those with *APOC3* LOF compared with nonvariant carriers matched for age and BMI. This resulted in substantial reductions in the circulating levels of these lipoprotein classes and in the time taken for large VLDL particles to transit down the delipidation cascade (from VLDL_1_ to LDL). Third, a potentially significant finding from a mechanistic viewpoint was that the rate of direct clearance of VLDL (especially VLDL_1_) from the circulation was higher in the *APOC3* LOF carriers. Fourth, it was noteworthy that having a lower apoC-III level was not associated with altered production rates for VLDL or chylomicrons, nor did it affect the production or clearance rate of LDL, which explains why total plasma apoB and LDL-C were the same in *APOC3* LOF carriers as in nonvariant carriers. The findings of this study have implications for treatment strategies directed toward apoC-III as an intervention target (17, 32–37).

The results in this group of unrelated patients (5 of whom were heterozygous for a likely previously unstudied *APOC3* LOF variant) are in line with, and build on, the metabolic studies conducted in the Amish population, where carriers of a null mutation in *APOC3* (R19X) were compared with unaffected siblings. In the investigation by Reyes-Soffer et al. (31), the mutation in *APOC3* was associated with approximately 50% lower plasma apoC-III levels, 35% lower plasma TG, and 36% lower total VLDL apoB. There was no difference in VLDL production rates between the *APOC3* LOF carriers and their unaffected siblings, and the lower VLDL (and TG) level was attributed to an increased conversion rate to IDL and LDL. While a trend to increased direct clearance of VLDL apoB was seen in this earlier study, it was not significant. We were able to establish that lack of apoC-III did substantially affect the delipidation rates of both chylomicrons and VLDL and provided evidence for a significant effect (*P* = 0.015) on apoB100-containing VLDL direct clearance. The observation that our *APOC3* LOF carriers had significantly lower (*P* = 0.037) apoC-III production rates establishes the nonsignificant trend seen in the Amish study (31). It is likely that decreased production of apoC-III is a consequence of the genetic variation in these 2 reports and that the significantly increased (*P* = 0.010) FCR for this apolipoprotein seen in our study is due to the more rapid clearance of TRL particles containing this protein on their surface rather than a direct result of the LOF mutation. The data from these highly informative studies in genetically defined patients, combined with metabolic investigations across participant groups with a wide range of TG levels that showed a strong, inverse association between high plasma apoC-III levels and lower VLDL and chylomicron clearance rates, lead to the conclusion that this apolipoprotein is a major regulator of TRL lipolysis and a suitable target for intervention (1, 4, 13, 18, 23). Examination of these rare individuals with LOF variants not only reduced the potential for confounding factors to influence interpretation of the kinetic findings but also demonstrated vividly the large differences in TRL metabolism that accompany *APOC3* LOF even in the heterozygous state. The observation that the clearance rate of LDL apoB100 (and hence LDL particles) was unaffected by genetically determined low apoC-III levels in both the present and previous investigations (31) indicates that apoC-III has no impact on LDL receptor activity and provides a possible explanation as to why apoE kinetics did not differ between *APOC3* LOF carriers and noncarriers (apoE and apoB100 are the major ligands for the LDL receptor) (17).

Given the substantial impact that reduced apoC-III levels had on the metabolism of intestinally derived chylomicrons and liver-derived VLDL, the question arises as to what physiological role this protein has in people with normal or optimal TG levels (17). One possibility is that apoC-III has a counterregulatory action, which moderates the degree of TRL lipolysis in a tissue bed, for example, cardiac or skeletal muscle. In our evolutionary past, access to fat-rich foods, especially animal-based foods, was likely to be episodic and required the expenditure of energy over a prolonged period (to meet the challenge of hunting prey). LPL appears to be a high-capacity, very efficient enzyme for removing TG from the core of TRL particles, and potentially an inhibitor present on TRL may operate to ensure delivery to multiple tissue sites rather than have the full TG load in a chylomicron or VLDL_1_ particle removed at “first pass.” *APOC3* LOF variants are rare in populations, and this may be due to the requirement to have, when fat in the diet is scarce (as it has been throughout much of human history), a prolonged circulation time for TRL — of the order of near 30 minutes for chylomicrons and about 12 hours for VLDL compared with the less than 8 minutes and 3 hours, respectively, seen when apoC-III is very low.

The major limitation of this investigation relates principally to the rarity of *APOC3* LOF variant carriers in the population. We had to screen large numbers of genetically defined individuals to identify our patients whereas the previous study relied on an isolated population cohort where rare variants may be enriched (31). It follows, therefore, that the number of LOF carriers is small, and we have to accept a sex imbalance relative to the noncarrier group. We believe this did not significantly affect the interpretation of the kinetic results since the differences between the groups were so marked.

Genetic studies have provided convincing evidence that *APOC3* loss of function even in the heterozygous state is associated with reduced risk of ASCVD (5–9, 38). This protective effect may be attributable to TG-lowering consequences of having a low apoC-III, or according to some studies, apoC-III may have a more direct atherogenic action (1, 39–41). The observation in studies of apoB metabolism in *APOC3* LOF and other investigations (7, 8) that low levels of apoC-III are not linked to reduction in total plasma apoB or LDL-C prompts consideration of this apolipoprotein’s role in determining the abundance of remnant lipoproteins in the circulation. These partially lipolyzed TRLs are cholesterol rich and are thought to have an atherogenic potential that is equal to or exceeds that of LDL (17–21). In the present study, we did observe substantially lower concentrations of VLDL_1_, VLDL_2_, and IDL (likely associated with decreased levels of apoB100-containing remnants) and also plasma apoB48 (a marker of chylomicron remnant concentration) in the group of *APOC3* LOF carriers. Whether this reduced abundance of remnants is sufficient to account fully for the decrease in ASCVD risk seen in *APOC3* LOF carriers is yet to be determined. Finally, it is worthwhile to compare the findings of studies in patients with *APOC3* LOF variants associated with reduced apoC-III production to the results of clinical trials of agents designed to suppress apoC-III synthesis. Olezarsen, an antisense oligonucleotide drug that interferes with *APOC3* mRNA translation, was found at the highest dose to reduce apoC-III by 74%, plasma TG by 60%, and plasma apoB by 10% but LDL-C was largely unaffected (32). The accord between the naturally occurring mutations and pharmacological intervention is striking. Previous in vitro observations have suggested a role for apoC-III in the regulation of VLDL assembly and secretion (1, 2, 28). However, the lack of effect of *APOC3* LOF variants on CM-apoB48 and VLDL apoB100 production rates indicates that this finding may not translate to the human situation. The *APOC3* LOF variants would likely affect intracellular (intrahepatic) apoC-III content as well as the circulating concentration of the apoprotein.

In conclusion, in a group of unrelated patients studied in the physiological setting of postprandial lipemia, we found that *APOC3* LOF variants were associated with dramatic and highly specific changes in TG metabolism. Lipolysis rates were increased several-fold and plasma concentrations of TRL and IDL decreased as a consequence. There was no impact on production rates of apoB48-containing lipoproteins or apoB100-containing lipoproteins, which is an important observation indicating that use of lipid-lowering agents directed at apoC-III may not impair TG export from liver and intestine. The observation that reducing apoC-III levels was associated with a rapid transit of TRL particles down the delipidation pathway suggests that the rate of remnant formation will be diminished, and further, the finding that direct clearance of VLDL was higher when apoC-III was low indicates that remnant removal may be enhanced. The net effect of genetically determined lower apoC-III on ASCVD risk is well documented; however, how this is linked to the substantial perturbations in TRL metabolism given that LDL levels are not altered is yet to be defined.

## Methods

### Participants.

This study was performed in 12 patients (White, of Finnish ancestry): 6 (3 men and 3 postmenopausal women) were heterozygotes for *APOC3* LOF mutations (5 for rs138326449 and 1 for rs7653203), and 6 controls (all men) were noncarriers of any known *APOC3* LOF variant and matched for age and BMI with the carriers. The 6 *APOC3* variant carriers were identified initially from previously genotyped and whole exome–sequenced participants in the THL Biobank in Finland (https://thl.fi/en/web/thl-biobank/for-researchers/sample-collections), and their genotypes were subjected to confirmatory analysis. The noncarriers were selected from previous kinetic study cohorts (42–44). General inclusion criteria were age 20 to 75 years, BMI less than 35 kg/m^2^, and nonsmoking status. Exclusion criteria were a history of any cardiovascular or severe disease, any condition affecting lipid levels, abnormalities in thyroid or kidney function, or hematological abnormalities. None of the participants used any medication or hormones known to influence lipid metabolism.

### Metabolic study protocol.

Patients were admitted to the clinical research unit at the Helsinki University Central Hospital after a 12-hour fast at 8:00 am. To examine the kinetics of apoB48, apoB100, apoC-III, and apoE, we used a tracer of deuterated leucine (5,5,5-D3 Euriso-Top, D3-leucine) at a dose of 7 mg/kg body weight. To examine the kinetics of TG, we used a tracer of deuterated glycerol (D1,1,2,3,3, D5 Euriso-Top) at a dose of 500 mg (44, 45). Two hours after tracer administration, a standard fat-rich meal (927 kcal) comprising bread, butter, cheese, ham, boiled egg, fresh red pepper, low-fat (1%) milk, orange juice, and tea or coffee (63 g carbohydrate, 69 g fat, and 40 g protein) was consumed within 10 minutes. Blood samples were drawn before tracer injection and at frequent intervals thereafter until 10 hours postadministration, when a dinner was served. The patients remained physically inactive and only water was drunk (ad libitum) during the study. The patients returned the following morning again in the fasting state to give blood at 24 hours after tracer administration. In addition, the participants came back fasting at 8:00 am on days 2, 3, 4, and 7 to allow measurements of D3-leucine enrichments of apoB100 in IDL and LDL.

### Tracer enrichment in apolipoproteins and TGs, multicompartmental modeling, and parameter estimation.

The protocol for kinetic investigation and the compartmental model structure for apoB48/apoB100/TG has been described in detail previously (27, 44–46). The experimental protocol and modeling for apoC-III and apoE have also been reported (27, 46). Modeling and parameter estimation were performed using SAAMII (47).

### Lipoprotein isolation and biochemical analyses.

Chylomicrons, VLDL_1_ (Svedberg flotation coefficient 60–400), and VLDL_2_ (Svedberg flotation coefficient 20–60) were isolated from blood samples by density gradient centrifugation (48), and IDL and LDL were prepared using sequential fixed density centrifugation (46). Concentrations of TGs and cholesterol in total plasma and lipoprotein fractions were analyzed using the Konelab 60i analyzer (Thermo Fisher Scientific). Other laboratory tests were performed using standard methods. Total plasma and postprandial apoB48 levels were measured by ELISA (Shibayagi). Plasma levels of apoC-III were determined with an immunoturbidimetry-based method (Kamiya Biochemical Company). ELISAs were used to measure serum ApoE (STA-367, Cell Biolabs), ANGPTL3 (DANL30, R&D Systems, Bio-Techne), and ApoA5 (LS-F5818, LifeSpan Biosciences).

### Lipidomic analysis.

Lipids were extracted from the lipoprotein fractions using the BUME method (49). The lipid extracts were diluted in chloroform/methanol (1:2) with 5 mM ammonium acetate, and the lipids were quantified by direct infusion (shotgun) analysis on a QTRAP 5500 mass spectrometer (SCIEX) equipped with a robotic nanoflow ion source, TriVersa NanoMate (Advion). The analysis of TGs was performed in positive ion mode by neutral loss detection of 10 common acyl fragments formed during collision-induced dissociation according to previous work (50). The phosphatidylcholines were also detected in positive mode using precursor ion scanning of *m/z* 184.1 as described previously (51). Glyceryl-d_5_-hexadecanoate (CDN Isotopes) and diheptadecanoyl (17:0/17:0) phosphatidylcholine were added during the extraction and used for quantification.

### Lipase measures.

A bolus injection of 75 IU/kg heparin was injected intravenously after an overnight fast to release lipase in the circulation. An immunochemical method was used to measure selectively lipoprotein and hepatic lipase activities in postheparin plasma isolated from blood samples taken 10 minutes after the heparin bolus (52).

### Determination of intra-abdominal fat depots.

Subcutaneous abdominal and intra-abdominal fat content were determined using a clinical 1.5 Tesla magnetic resonance imager (MAGNETOM, Avanto, Siemens) (53). Patients were advised to fast for 4 hours before imaging.

### Statistics.

All statistical analyses were performed using R (version 4.0.2). *P* values for baseline characteristics in Table 1 were calculated using a *t* test for continuous variables and a χ^2^ test for categorical variables (as per standard settings in R package tableone). *P* values for kinetic parameters (Tables 2 and 3) and lipidomics (Supplemental Figure 2, A and B) were calculated using the Mann-Whitney *U* test using the wilcox.test() function in R. *P* < 0.05 was considered statistically significant.

### Study approval.

The study design was approved by the Ethics Committee II of Helsinki University Central Hospital, Helsinki, Finland (ClinicalTrials.gov NCT04209816 and NCT01445730). The study was performed in accordance with the Declaration of Helsinki and the European Medicines Agency note for guidance on good clinical practice. All study participants gave written informed consent before any study procedures were initiated.

## Author contributions

The authors contributed to the present work as follows: JB, NM, and MRT contributed to conception and design; EB, NM, SS, JR, MMA, AH, CS, AT, LA, POB, MH, SR, MA, SR, and ML to the acquisition of data or analysis; and JB, CJP, MRT, EB, and MA to the interpretation of data. JB, CJP, MRT, EB, and MA drafted the original and revised manuscripts, and all authors approved the final version to be published.

## Supplementary Material

Supplemental data

ICMJE disclosure forms

## Figures and Tables

**Table 1:**
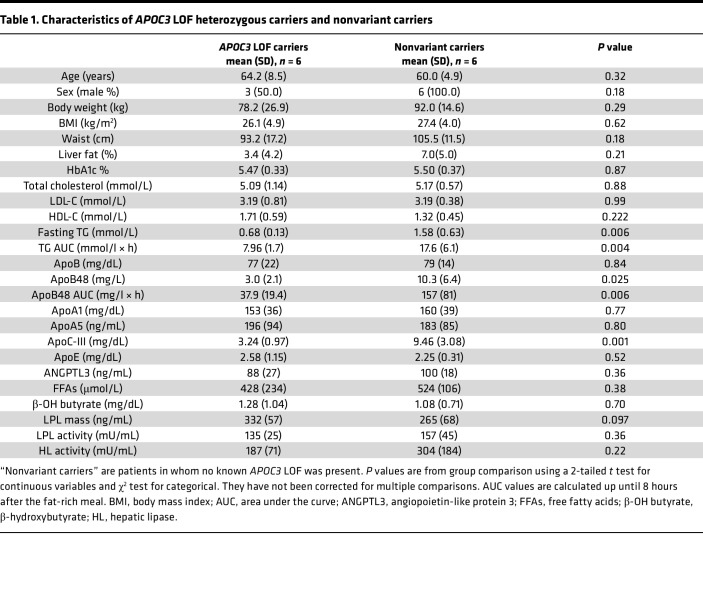
Characteristics of *APOC3* LOF heterozygous carriers and nonvariant carriers

**Table 2:**
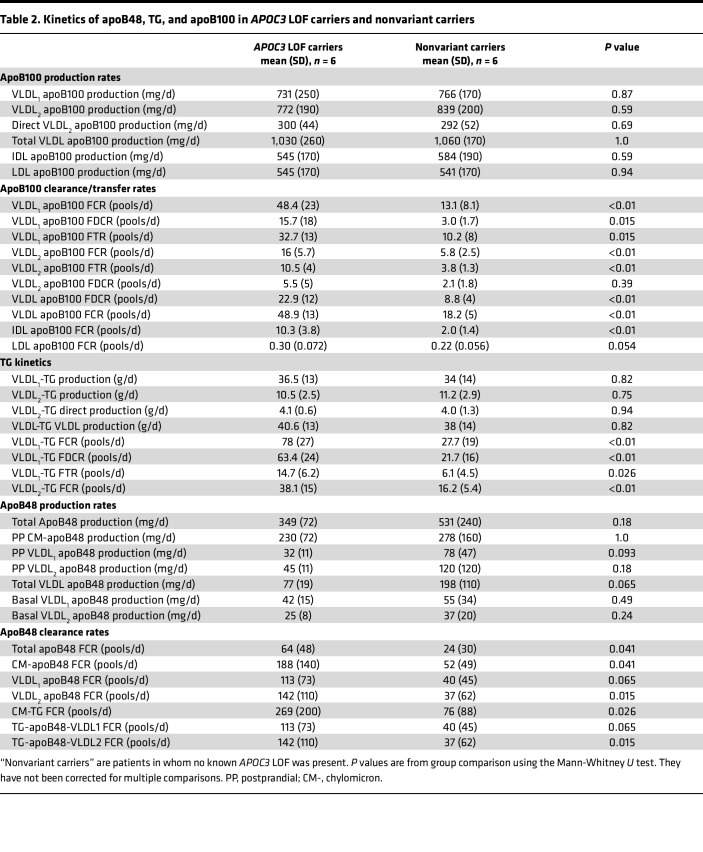
Kinetics of apoB48, TG, and apoB100 in *APOC3* LOF carriers and nonvariant carriers

**Table 3:**
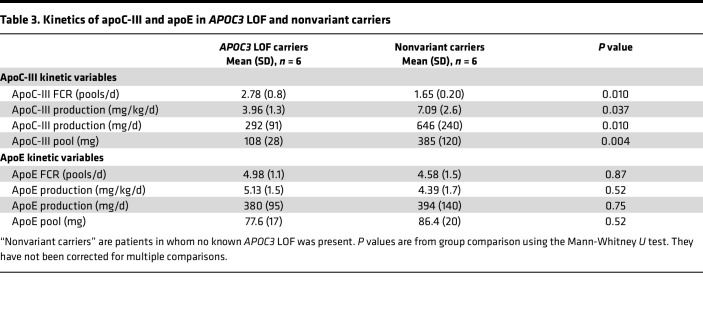
Kinetics of apoC-III and apoE in *APOC3* LOF and nonvariant carriers

## Version 1 08/30/2022

In-Press Preview

## Version 2 10/10/2022

Electronic publication
