# Supplementary material for: Postprandial metabolism of apolipoproteins B48, B100, C-III, and E in humans with APOC3 loss-of-function mutations
Source: JCI Insight. 2022 Oct 10;7(19):e160607. doi: 10.1172/jci.insight.160607 (PMC9675484; doi:10.1172/jci.insight.160607)

SUPPLEMENTAL FILE

Postprandial metabolism of apolipoproteins B48, B100, C-III and E in in humans with *APOC3* loss-of-function mutations

Taskinen MR, Björnson E, Matikainen N, Söderlund S, Rämö J, Ainola M, Hakkarainen A, Sihlbom C, Thorsell A, Andersson L, Bergh P-O, Henricsson M, Romeo S, Adiels M, Ripatti S, Laakso M, Packard CJ* and Borén J*

* shared last authors

Supplementary Table 1

Additional group characteristics for *APOC3* LOF and non-variant carriers.

	APOC3 LOF carriers Mean (SD)	Non-variant carriers Mean (SD)	P-value	*APOC3* LOF carriers Median [range]	Non-variant carriers Median [range]	P-value
N	6	6		6	6	
Age (years)	64.2 (8.47)	60.0 (4.94)	0.32	65.5 [48.0, 71.0]	60.0 [51.0, 65.0]	0.105
Gender	3 (50.0 %)	6 (100.0)	0.18	3 (50.0)	6 (100.0)	0.18
Body weight (kg)	78.2 (26.9)	92.0 (14.6)	0.29	71.1 [46.0, 120.0]	94.8 [69.6, 112.7]	0.34
BMI (kg/m^2)	26.1 (4.9)	27.4 (4.03)	0.62	25.3 [20.2, 34.0]	27.8 [20.5, 33.1]	0.52
Waist (cm)	93.2 (17.2)	105.5 (11.5)	0.18	89.0 [73.0, 118.0]	105.2 [89.0, 124.5]	0.26
Syst. BP (mmHg)	138.2 (25.2)	131.0 (14.3)	0.56	126.0 [116.0, 180.0]	131.0 [111.0, 152.0]	0.75
Diast. BP (mmHg)	79.2 (7.2)	82.8 (4.1)	0.30	81.5 [67.0, 88.0]	84.0 [77.0, 88.0]	0.29
Glucose (mmol/l)	5.9 (0.67)	5.7 (0.30)	0.49	5.8 [5.4, 7.0]	5.6 [5.5, 6.3]	0.87
Insulin (mU/l)	8.9 (7.5)	7.8 (4.3)	0.77	5.5 [2.8, 19.4]	7.4 [3.0, 14.4]	0.75
HbA1c (mmol/mol)	36.2 (3.8)	36.5 (4.2)	0.89	36.0 [30.0, 40.0]	36.5 [31.0, 41.0]	0.75
HbA1c (%)	5.5 (0.33)	5.5 (0.37)	0.87	5.4 [4.9, 5.8]	5.5 [5.0, 5.9]	0.75
Tot chol (mmol/l)	5.1 (1.1)	5.2 (0.57)	0.88	5.3 [3.2, 6.5]	5.1 [4.5, 5.8]	1.00
LDL-chol (mmol/l)	3.2 (0.81)	3.2 (0.38)	1.0	3.1 [2.1, 4.3]	3.2 [2.5, 3.6]	0.87
HDL-chol (mmol/l)	1.7 (0.59)	1.3 (0.45)	0.22	1.7 [0.9, 2.4]	1.4 [0.6, 1.8]	0.26
Fasting TG (mmol/l)	0.68 (0.13)	1.58 (0.63)	0.006	0.65 [0.51, 0.88]	1.6 [0.78, 2.2]	0.008
ApoB (mg/dl)	76.9 (21.6)	79.1 (14.2)	0.84	73.3 [54.0, 105.0]	84.1 [56.5, 95.1]	0.75
ApoA1 (mg/dl)	153.5 (36.3)	160.2 (39.0)	0.77	150.4 [113.1, 212.8]	160.5 [102.0, 207.0]	0.87
ApoC-III (mg/dl)	3.2 (0.97)	9.5 (3.08)	0.001	3.6 [1.4, 4.1]	9.9 [4.1, 12.9]	0.004
FFA (µmol/l)	428.4 (234.2)	524.0 (105.8)	0.38	373.3 [193.0, 855.0]	508.0 [362.0, 671.0]	0.11
β-OH butyrate (mg/dl)	1.28 (1.04)	1.08 (0.71)	0.70	0.94 [0.46, 3.22]	0.82 [0.55, 2.37]	1.00
ApoB48 ug/ml	3.00 (2.12)	10.34 (6.35)	0.025	2.93 [0.36, 6.38]	8.33 [5.45, 20.96]	0.018
LPL-activity (mU/ml)	135.2 (25.4)	156.9 (44.9)	0.36	128.0 [109.0, 163.0]	159.5 [90.7, 207.0]	0.27
HL-activity (mU/ml)	187.4 (70.6)	304.4 (184.4)	0.22	151.0 [124.0, 297.0]	240.5 [92.3, 591.0]	0.36
LPL-mass (ng/ml)	331.8 (56.7)	265.4 (68.5)	0.10	328 [270, 412]	257 [190, 388]	0.055
Liver fat (%)	3.4 (4.2)	7.0 (5.0)	0.21	1.6 [0.2, 11.0]	5.9 [1.1, 13.8]	0.11
VAT area (cm^2)	1515 (1262)	2781 (1199)	0.10	1294 [332, 3173]	2729 [1191, 4711]	0.15
SAT area (cm^2)	2457 (859)	3361 (701)	0.07	2441 [1301, 3907]	3291 [2454, 4601]	0.078
ALT (U/l)	19.2 (11.7)	31.8 (10.8)	0.08	18.0 [9.0, 41.0]	29.0 [24.0, 53.0]	0.036
ASAT (U/l)	24.3 (7.3)	32.0 (5.1)	0.06	24.5 [16.0, 35.0]	31.0 [28.0, 42.0]	0.063
AFOS (U/l)	80.7 (34.8)	48.0 (12.4)	0.06	69.5 [59.0, 151.0]	47.5 [27.0, 61.0]	0.010
GT (U/l)	32.2 (26.7)	31.2 (9.2)	0.93	20.5 [15.0, 85.0]	29.0 [24.0, 49.0]	0.26
ApoC-III FCR (pools/day)	2.78 (0.78)	1.62 (0.20)	0.01	2.66 [1.74, 4.04]	1.67 [1.31, 1.85]	0.011
ApoC-III SR (mg/kg/day)	3.96 (1.32)	7.03 (2.85)	0.04	3.88 [2.49, 5.75]	7.44 [3.83, 10.39]	0.068
ApoC-III SR (mg/day)	291.5 (90.9)	672.5 (257.1)	0.008	279.6 [192.4, 446.9]	838.9 [359.2, 880.3]	0.018
ApoC-III pool	3.21 (0.82)	9.44 (3.05)	0.001	3.16 [2.04, 4.61]	9.90 [6.36, 13.56]	0.006
ApoE mg/dl	2.58 (1.15)	2.25 (0.31)	0.52	2.29 [1.42, 4.09]	2.34 [1.81, 2.60]	1.00
ANGPTL3 ng/ml	87.6 (26.6)	100.1 (17.9)	0.36	75.9 [71.5, 140.7]	92.1 [84.1, 125.8]	0.078

P-values in columns three have been calculated using a t-test, and p-values in column six have been calculated using the Mann-Whitney U test. SAT, subcutaneous adipose tissue; VAT, visceral adipose tissue.

Supplementary Table 2

Plasma concentrations of apoE, apoA5, apoC-III and Angptl3 prior to administration of standard fat meal (0 hours) and 4 and 8 hours after consumption of meal.

	***APOC3* LOF carriers** mean (SD), n=6			**Non-variant carriers** mean (SD), n=6		
	0 h	4 h after meal	8 h after meal	0 h	4 h after meal	8 h after meal
ApoE	2.58 (1.15)	2.3 (0.89)	2.47 (1.29)	2.25 (0.31)	2.33 (0.18)	2.35 (0.54)
ApoA5	196 (94)	221 (92)	194 (78)	183 (85)	248 (77)	202 (92)
ApoC-III	3.67 (0.89)	2.89 (0.94)	3.22 (0.86)	10.48 (2.19)	10.51 (3.05)	9.74 (2.98)
Angptl3	87.6 (26.5)	89.4 (33.4)	81.9 (31.9)	100.1 (17.9)	103.8 (15)	85.9 (20.7)

Data is mean (SD), Units are mg/dl for apoE and apoCIII and ng/ml for apoA5 and Angptl3. There were no statistical differences between *APOC3* LOF carriers and non-variant carriers at any time-point.

Supplementary Table 3

Fluxes and pool sizes for *APOC3* LOF and non-variant carriers.

	***APOC3* LOF**<br>**Mean (SD)**	**Non-variant carriers**<br>**Mean (SD)**	**P-value**
VLDL1 pool size (mg)	19.1 (20)	74.1 (30)	0.0087
VLDL2 pool size (mg)	50.5 (10)	165 (80)	0.0022
VLDL pool size (mg)	69.6 (20)	239 (90)	0.0022
IDL pool size (mg)	61.3 (30)	354 (200)	0.0087
LDL pool size (mg)	1880 (700)	2520 (600)	0.2
Flux into VLDL1 (mg/d)	730 (250)	766 (170)	0.87
Flux out from VLDL1 (mg/d)	259 (260)	218 (180)	0.82
Flux VLDL1 to VLDL2 (mg/d)	473 (160)	547 (170)	0.49
Flux into VLDL2 (mg/d)	773 (190)	840 (200)	0.59
Flux out from VLDL2 (mg/d)	254 (210)	271 (120)	0.82
Total flux out from VLDL (mg/d)	511 (300)	490 (160)	0.82
Flux VLDL2 to IDL (mg/d)	518 (170)	569 (190)	0.59
Direct IDL secretion (mg/d)	27 (14)	15 (12)	0.19
Flux into IDL (mg/d)	545 (170)	583 (190)	0.59
Flux out from IDL (mg/d)	0	42.5 (86)	0.0096
Flux IDL to LDL (mg/d)	545 (170)	542 (170)	0.94
Direct LDL secretion (mg/d)	166 (57)	68 (31)	0.013
Flux into LDL (mg/d)	710 (170)	610 (170)	0.39
Flux out from LDL (mg/d)	710 (170)	610 (170)	0.39

Data are presented as mean and standard deviation (SD). 'Non-variant carriers' are subjects in whom no known *APOC3* LOF was present. P values are from group comparison using the Mann-Whitney U test. They have not been corrected for multiple comparisons.

Supplementary Figure 1

Tracer kinetics in *APOC3* LOF and non-variant carriers. Turquoise colour refers to the non-variant carriers and red colour refers to the *APOC3* LOF carriers. Points shows mean experimental values and vertical dashes shows standard deviations. Solid line refers to the mean of the model fits.

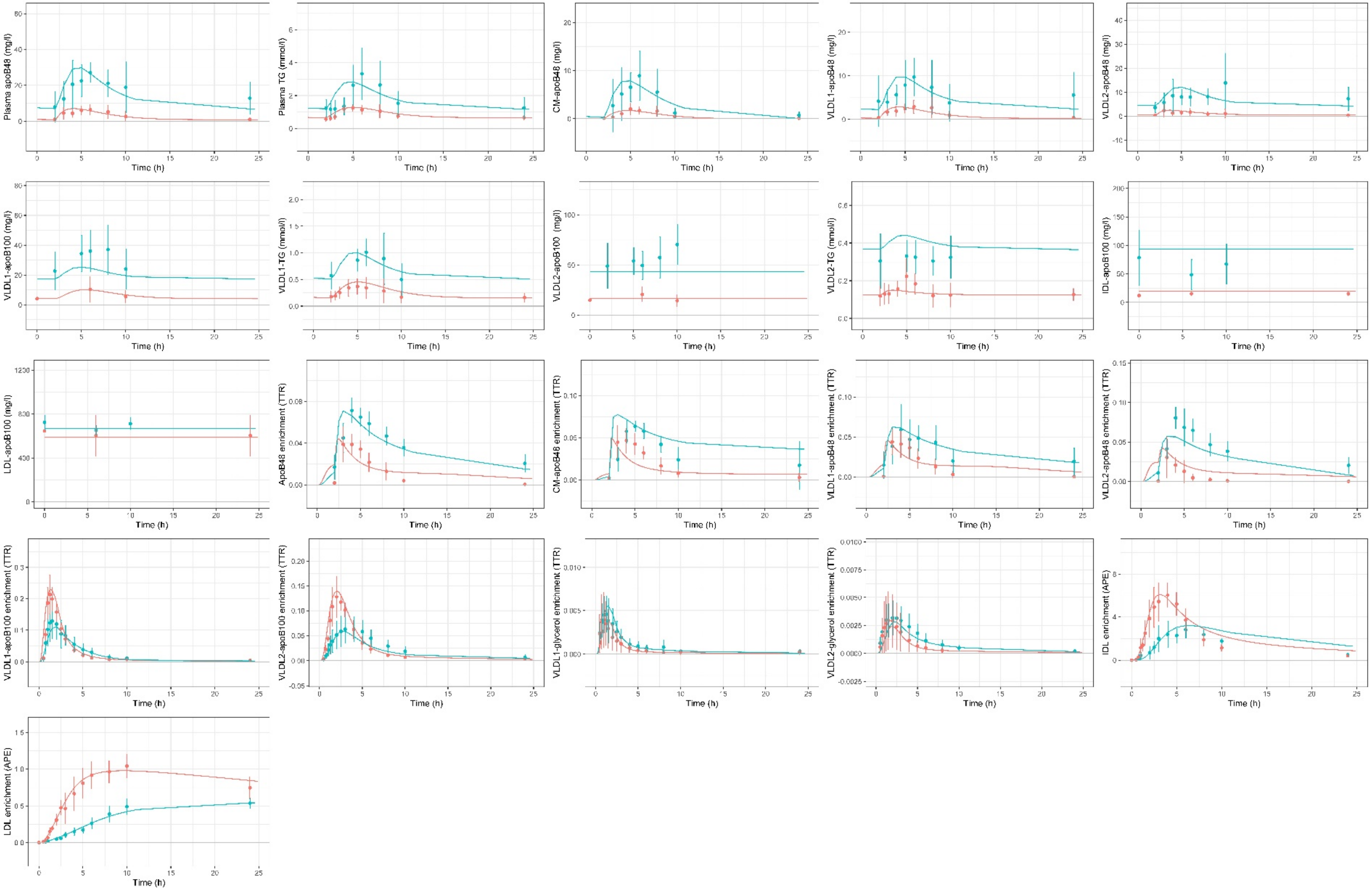

Supplementary Figure 2A

Relative abundance of triglyceride species within lipoprotein fractions. Turquoise colour refers to the non-variant carriers and red colour refers to the *APOC3* LOF carriers. No comparison between *APOC3* LOF carriers and non-carriers achieved a significance level below alpha=0.01. The relative abundance of each lipid species is expressed as molar % of total.

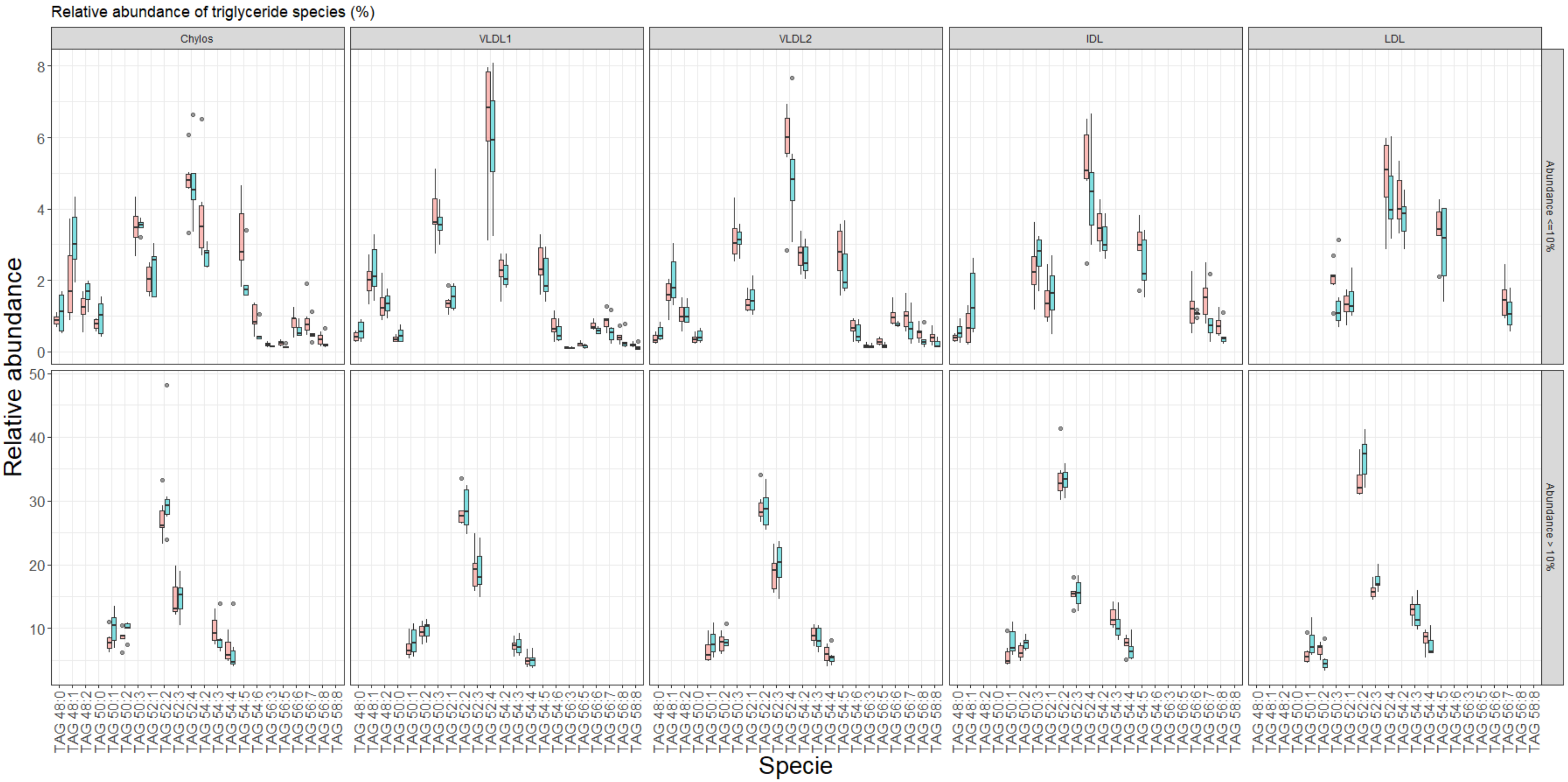

Supplementary Figure 2B

Relative abundance of triglyceride species within lipoprotein fractions. Turquoise colour refers to the non-variant carriers and red colour refers to the *APOC3* LOF carriers. At the level of alpha=0.01 only four species were significant: $VLDL_1$ PC 34:2 and PC 40:7, and IDL PC 34:4 and IDL PC 40:5. The relative abundance of each lipid species is expressed as molar % of total.

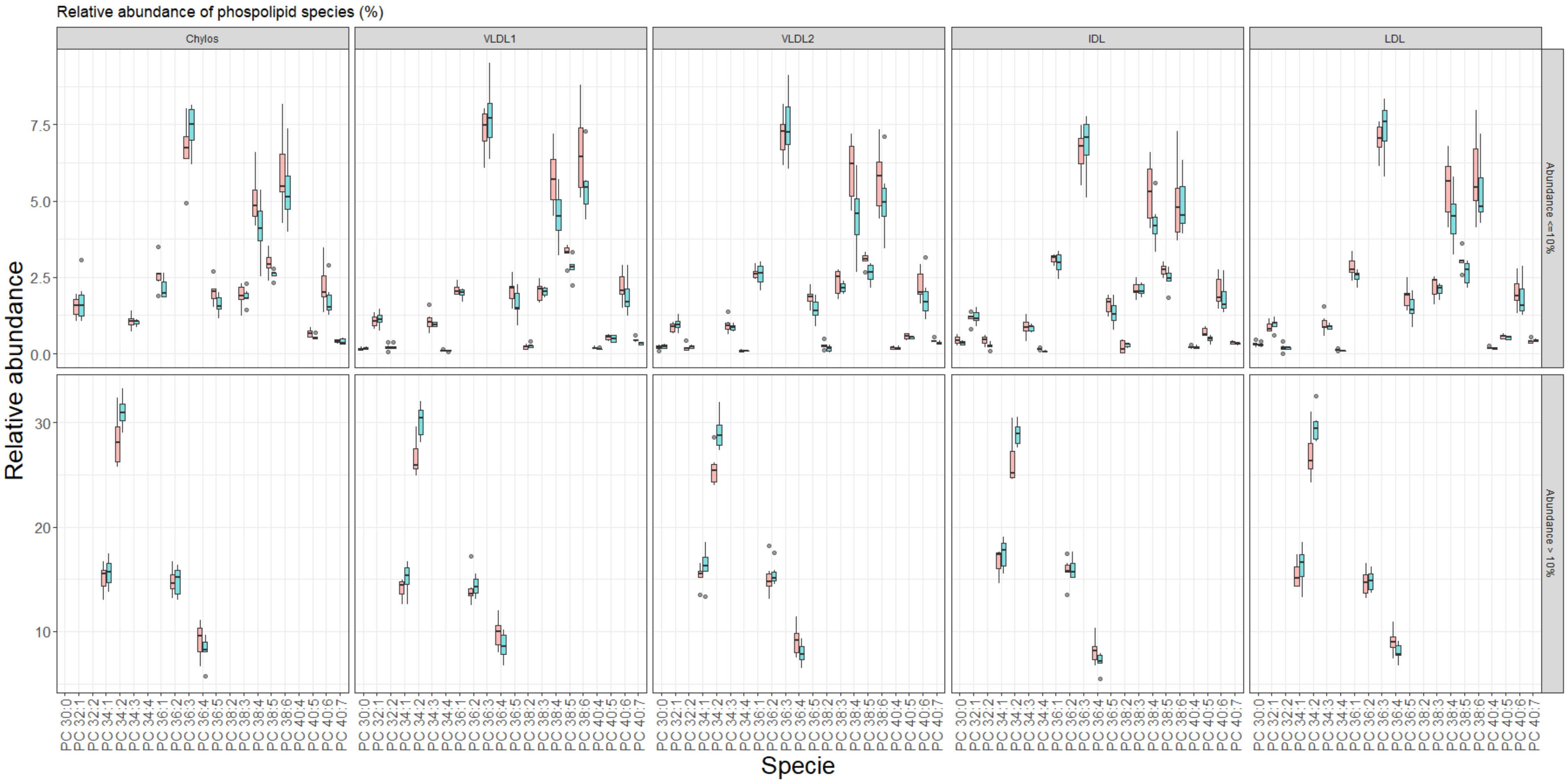

Supplement: Supplemental data [file jciinsight-7-160607-s237.pdf]
